# Who Packed the Drugs? Application of Bayesian Networks to Address Questions of DNA Transfer, Persistence, and Recovery from Plastic Bags and Tape

**DOI:** 10.3390/genes13010018

**Published:** 2021-12-22

**Authors:** Ane Elida Fonneløp, Sara Faria, Gnanagowry Shanthan, Peter Gill

**Affiliations:** 1 Department of Forensic Sciences, Oslo University Hospital, 0372 Oslo, Norway; rmgnsa@ous-hf.no (G.S.); peterd.gill@gmail.com (P.G.); 2 Faculty of Sciences, University of Lisbon, 1749-016 Lisbon, Portugal; sarafaria1998@outlook.pt; 3 Department of Forensic Medicine, University of Oslo, 0315 Oslo, Norway

**Keywords:** transfer, persistence, activity, Bayesian networks

## Abstract

When DNA from a suspect is detected in a sample collected at a crime scene, there can be alternative explanations about the activity that may have led to the transfer, persistence and recovery of his/her DNA. Previous studies have shown that DNA can be indirectly transferred via intermediate surfaces and that DNA on a previously used object can persist after subsequent use of another individual. In addition, it has been shown that a person’s shedder status may influence transfer, persistence, prevalence, and recovery of DNA. In this study we have investigated transfer persistence and recovery on zip-lock bags and tape, which are commonly encountered in drug cases and how the shedder status of the participants influenced the results. A probabilistic framework was developed which was based on a previously described Bayesian network with case-specific modifications. Continuous modelling of data was used to inform the Bayesian networks and two case scenarios were investigated. In the specific scenarios only moderate to low support for *H_p_* was obtained. Applying a continuous model based on the profile quality can change the LRs.

## 1. Introduction

In criminal cases, when DNA from a suspect is detected in samples collected from drug wrappings, there can be alternative explanations about the activity that may have led to the transfer, persistence, and recovery of his/her DNA. Often these explanations involve indirect transfer from an object they have used (e.g., bag or clothing), or perhaps the suspect has been in contact with the object in a situation not related to the crime. The hierarchy of propositions describes the different levels of evaluation of the evidence given propositions at sub-source, source, activity, and offence level [1,2,3]. The guidelines from the International Society for Forensic Genetics (ISFG) DNA commission provide advice on evaluation and the formulation of propositions and recommend the evaluation of results at activity level in the light of the alternative propositions of the case and calculating a likelihood ratio (LR) [2]. A recommended tool to aid in the evaluation of evidence is Bayesian networks (BNs) [4] and examples of how they can be constructed and used are widely available [5,6]. To construct a BN, the relevant variables and their respective probabilities are required. Extensive research on transfer persistence, prevalence, and recovery (TPPR) of DNA has been conducted over the past 10 years [7], although knowledge bases that can be utilised for routine casework are currently lacking.

The indirect transfer of DNA is dependent upon several variables including: the amount of DNA present, time since initial contact, number of contacts and the type of surface of the objects involved [8,9,10]. DNA from a person who previously handled an object can be detected for some time after the object has been handed to a new user [10]. However, it is more likely that DNA from the most recent user of the object will become the major contributor over time [11,12]. Van Oorschot et al. [12] observed an average 50% drop in the DNA from the previous user immediately after handling by a new user when the surface was hard and non-porous. Mayuoni-Kirshenbaum et al. [13] studied the persistence of DNA from a person cutting aluminium foil when the foil was subsequently used for lock picking by another individual and only detected DNA from the cutter in 1/25 cases while the lock-picker was detected in 9/25 samples.

It has previously been demonstrated that individuals have a different propensity to transfer DNA to handled items (shedder status); shedder status is consistent over years, and it can influence transfer probabilities [14,15,16]. However, the classification method has been debated and a need to introduce an intermediate shedder category (between high and low) has been demonstrated [15,16,17].

The aim of this paper is to provide a dataset along with a probabilistic framework to interpret DNA evidence at activity level. We present two specific scenarios related to DNA evidence in drug related cases. The first considers direct and indirect transfer to a zip-lock drugs bag that has been stored in a personal bag, whilst the second considers the persistence of DNA from a previous user of tape which was used to pack drugs by a second individual (the cardboard drug wrap experiment). The results from both experiments were examined in the light of the participant’s shedder status. A probabilistic framework was developed which was based on a previously described Bayesian network [5] with case-specific modifications. Previous work has usually applied discrete models to measure simple presence/absence of DNA that may be attributed to a person of interest (POI). A continuous model was demonstrated by Gill et al. [6], based upon sub-source likelihood ratios, and this was recently complemented with a method using mean $\bar{RFU}$ (peak height) instead of sub-source likelihood ratio [18]. Here we provide a demonstration of continuous modelling of indirect and direct transfer with two case examples. Continuous models are the preferred method with mixture analysis [19] because they take more information into account. It is natural that activity level assignments should take the same route. 

The paper is structured as follows: in Section 3.1,Section 3.2,Section 3.3 and Section 3.4 there is an empirical description of the data analysis, followed by probabilistic analysis using Bayesian networks of the zip-lock drugs bag experiment (Section 3.5.1), and the tape/cardboard drug wrap experiment (Section 3.5.2).

## 2. Materials and Methods

This project was approved by the data protection officer at Oslo University Hospital, all participants have given informed consent prior to participating in the experiments. Twenty participants were recruited and participated in all experiments.

### 2.1. Direct and Indirect Transfer to Zip-Lock Drugs Bags

Each participant was provided with a kit for the experiments containing two DNA-free (exposed to UV light for 10 min on each side) zip-lock bags (16.7 × 10 cm). One of the zip-lock bags contained a 15 mL centrifuge tube (VWR) filled with water to function as a weight.

#### 2.1.1. Direct Transfer

Participants were instructed to wait at least one hour after washing hands/using antibacterial hand wash, after which they touched the upper surface of the zip-lock bag for about 30 s by opening and closing it. They were then asked to repeat this process with the same bag two more times (total of three handlings). When the procedure was completed, participants were instructed to place the zip-lock bag into a new labelled envelope. The dataset generated is labelled E1 and is available in the Supplementary Material S2.

#### 2.1.2. Indirect Transfer

Participants were instructed to use their own personal bag/purse/backpack for this experiment. They were instructed to put on a pair of new disposable gloves before placing the zip-lock drugs bag containing the weight into their personal bag (which did not contain anything else during the experiment) and leave it there for 24 h; they were also instructed to move the personal bag during this period, e.g., carry it with them to work and home. After the 24 h had passed, the zip-lock drugs bag was removed, whilst wearing clean gloves, and placed into a new labelled envelope. The dataset generated is labelled E2 and is available in Supplementary Material S2.

### 2.2. Persistence and Detection of DNA from a Previous User of a Tape Roll

In this experiment participants were divided into pairs. Participant B was given a DNA-free (treated with UV light for 20 min on each side) roll of tape. The participants were instructed to wait at least 1 h after washing hands/using antibacterial liquid; then the tape was handled for 1 min by passing it from one hand to another, stroking the sides of the tape roll towards the palm of the hands; use of the tape was mimicked by tearing off a small piece. The roll of tape was placed in a labelled envelope and passed to participant A after a minimum 10 days in storage. Participant A was provided with a piece of clean cardboard (approx. 6 × 10 cm) (UV treated on each side for 20 min), to mimic a pack of drugs. He/she was instructed to wrap the piece of cardboard with the tape (previously handled by B). The “drug wrap” was placed in a new labelled envelope. Each of the 20 participants contributed twice to the experiments swapping the roles of A and B, giving a total of 20 wrapped cardboards. The data generated is available in Supplementary Material S2.

### 2.3. Shedder Status

Participants were asked to hold a plastic tube (15 mL centrifuge tube high performance tube, VWR) for 10 s in their dominant hand, at least one hour after washing hands/using antibacterial liquid. The experiments were repeated 3 times, with a new clean tube; there was a gap of at least three hours between each experiment. The data generated is available in Supplementary Material S2.

### 2.4. Sample Processing

Samples were collected with a moistened (one drop of water) swab (Tubed Sterile Dryswab, Medical Wire). The sampling was performed on: (a) zip-lock bags: the top 3 cm of the outside and inside (including lock) of the zip-lock bags; (b) wrapped cardboard: the full outside and edges of the tape around the cardboard; (c): tubes: the full body of the 15 mL centrifuge tubes (not lids). One negative control sample was collected from all items (tape, cardboard, zip-lock bag and tube). For the samples collected from zip-lock bags and wrapped cardboard, the tips of the swabs were cut, placed in 1.5 mL Eppendorf tube and stored at −20 °C until further processing to avoid degradation. Samples collected from tubes were stored in ventilated evidence bags to dry before the tips of the swabs were cut directly into single PCR-tubes for a direct PCR analysis.

Samples collected from zip-lock bags and wrapped cardboard were extracted by the 5% Chelex procedure [20] (no prior incubation with water) where 200 µL Chelex (Bio-Rad, Hercules, CA, USA) was added to swabs. All samples were quantified with PowerQuant (Promega, Madison, WI, USA) on the 7500 Real-Time PCR system (ThermoFisher, Waltham, MA, USA) using the manufacturers’ recommendations. PCR amplification was carried out using the PowerPlex Fusion 6C System kit (Promega, Madison, WI, USA) as recommended by the manufacturer (1 ng template, 25 µL reaction volume and 29 amplification cycles). Samples that had lower concentrations than the recommended template amount were amplified with the maximum template volume of 15 µL. For the shedder experiment, the PCR reaction mix was added directly to the tube containing the swab tips. Amplification was carried out using a Veriti 96-Well Thermal Cycler (ThermoFisher, Waltham, MA, USA). Samples were injected on the Applied Biosystems 3500 xL Genetic Analyzer at 1.2 kV for 24 s. The results were analysed using the GeneMapper ID-X Software version 1.6 (ThermoFisher, Waltham, MA, USA). The analytical threshold (AT) for alleles was set to 100 RFU.

### 2.5. Data Analysis

Results were analysed using R version 4.0.3 with the package tidyverse version 1.3.0. Likelihood ratios and mixture proportions were calculated using EuroForMix version 3.2.0 using propositions shown in in Equation (1), where *U* corresponds to unknown contributors, necessary to explain all alleles present. The average RFU ($\bar{RFU})$ for a contributor was calculated by dividing the total RFU (*RFU_tot_*) by the number (*n*) loci (23, except amelogenin), Equation (2). If the sample was a mixture, the contribution from the POI was found by multiplying $\bar{RFU}$ for the sample by the mixture proportion (*M_x_*). If a sample was diluted then the result was multiplied by a dilution factor (*d_l_*).

(1) $$LR=\frac{\mathrm{Pr}\left(E|H_{p}:POI+U_{1}\right)}{\mathrm{Pr}\left(E|H_{d}:U_{1}+U_{2}\right)}$$

(2) $${\bar{RFU}}_{POI}=M_{x}\times \frac{RFU_{tot}}{n}\times d_{l}$$

#### 2.5.1. List of Variables

We follow the notation used by Gill et al. [6] (Section 4), summarised here:

*t* is the probability of direct transfer, persistence and recovery of DNA from the POI (under *H_p_* only).
*t’* is the probability of direct transfer, persistence and recovery of DNA from an unknown contributor (under *H_d_* only).
b is the probability of background DNA, based on observations, and is applied under both *H_p_* and *H_d_*. Background DNA is present from unknown sources and unknown activities. It can be described as “foreign” (non-self). For further details we refer to Section 3.2 in [6].
s is the probability of transfer, persistence and recovery; in experiment 1, this is indirect transfer under *H_p_* and *H_d_*, and in experiment 2, it is direct transfer under *H_d_* only.
Suffixes are applied to described probabilities of an event given a particular contributor, e.g., *t_A_* refers to the probability of direct transfer, persistence and recovery of DNA from contributor A.

#### 2.5.2. Notation Relating to the Experiments

Experiment 1: zip-lock drugs bag

$E1_{dbag}$: Dataset from the zip-lock drugs bag; direct transfer

$E2_{pbag}$: Dataset from the personal bag experiment; indirect transfer

${\bar{RFU}}_{E1dbag}$: Lognormal distribution of mean RFU from $E1_{dbag}$ data; direct transfer

${\bar{RFU}}_{E2pbag}$: Lognormal distribution of mean RFU from $E2_{pbag}$ data; indirect transfer

Experiment 2: Tape/cardboard drug wraps

$C1_{pack}$: Dataset from drugs wrapper; direct transfer

$C2_{tape:}$ Dataser from the tape handler; direct transfer

${\bar{RFU}}_{C1pack}$: Lognormal distribution of mean RFU from $C1_{pack}$ data; direct transfer

${\bar{RFU}}_{C2tape}$: Lognormal distribution of mean RFU from $C2_{tape:}$ data; direct transfer

General

$\bar{RFU}$*_A_*: the observed $\bar{RFU}$ value from contributor A

$\bar{RFU}$*_B_*: the observed $\bar{RFU}$ value from contributor B

$\bar{RFU}$*_unknown_*: the observed $\bar{RFU}$ value from an unknown contributor

#### 2.5.3. Distribution Fitting

Log-normal distributions were fitted to the data using the R package *fitdistrplus* using function *lnorm.* The method is described in detail in Supplementary Material S1. The probability of background for experiment 1 was modelled using the same lognormal parameters calculated for indirect transfer data ${\bar{RFU}}_{E2pbag}$, as described in the Supplementary Material S1 (Section S4.4.1). To carry out sensitivity analysis, 1000× bootstraps (with replacement) were taken of datasets (Supplementary Material S2). For each bootstrap, a new set of log-normal parameters (mean log and SD log) were calculated using the *fitdistrplus* R package using the *lnorm* function. The probability distributions were used to substantiate the Bayesian networks described in Section 3.5.1 and Section 3.5.2. The programming of these networks was carried with R-code using the formulae described in Section 3.5.1 and Section 3.5.2.

## 3. Results

### 3.1. Direct and Secondary Transfer to Zip-Lock Drugs Bags

**Figure 1:**
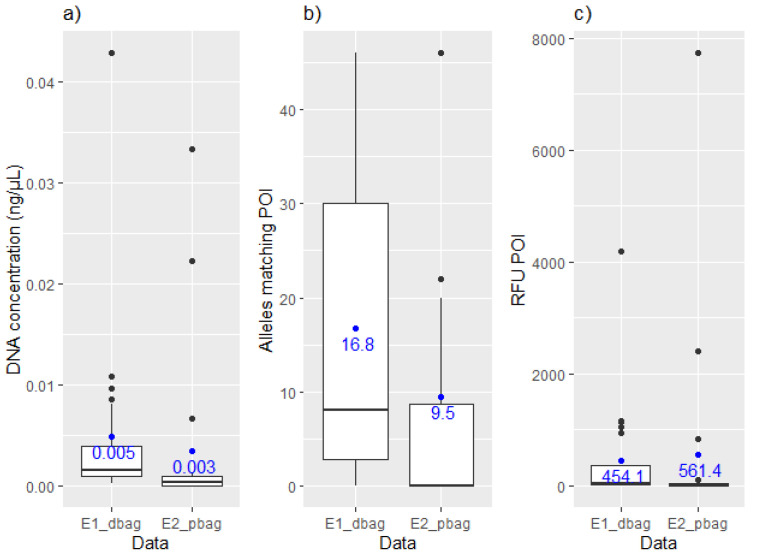
Boxplots displaying (**a**) the measured DNA concentrations (ng/µL in 200 µL), (**b**) the number of detected allele’s matching the POI and (**c**) the ${\bar{RFU}}_{POI}$ for the samples in the $E1_{dbag}$ (direct) and $E2_{pbag\;}$ (indirect) data.

The DNA from the POI could be detected in the samples collected after direct handling of a zip-lock drugs bag and after indirect transfer to the drugs bag from storage in a previously used personal bag. More alleles matching the POI along with higher DNA quantities were generally observed in the direct transfer experiment ($E1_{dbag}$) compared with indirect transfer from inside a personal bag ($E2_{pbag}$). Generally, the ${\bar{RFU}}_{POI}$ was higher in $E1_{dbag}$, but a few occurrences of higher ${\bar{RFU}}_{POI}$ were observed in $E2_{pbag}$ (Figure 1).

**Figure 2:**
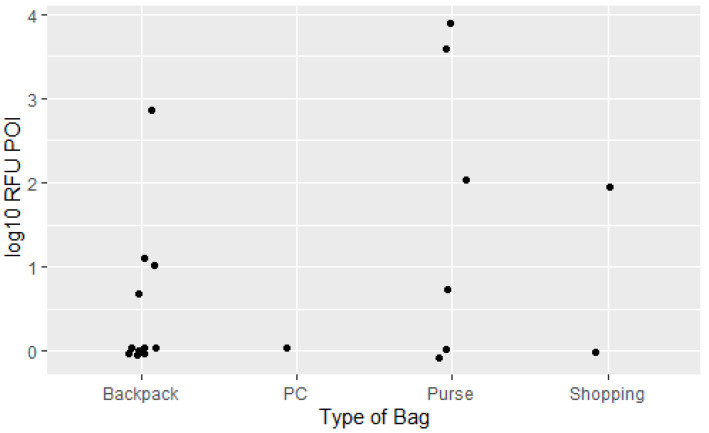
Dot plot displaying the log RFU¯10 values detected in the samples collected from zip-lock drugs bags after storage in a previously used personal bag (backpack, PC bag, purse/handbag or shopping bag).

In the direct transfer experiment four full and seven partial profiles were detected, while six samples only displayed six or less alleles and four samples had no results. Only one sample contained (two) unknown alleles. From the indirect transfer experiment three full and two partial profiles corresponding to the POI were detected. Four samples only displayed five or less alleles and ten samples had no profiling results. One sample was a mixture with 21 unknown alleles, and one sample was a partial profile with alleles corresponding to a child of the participant. The most frequently used type of personal bag used was a backpack but a large proportion of these samples (7/11) had no results. The full dataset can be found in the Supplementary Material S2. The highest $\bar{RFU}$ values on the zip-lock drugs bags were observed after storage in a personal purse/handbag, (Figure 2). A few participants (1 and 2, respectively) used a personal PC and shopping bag to store the zip-lock drugs bags. Most participants reported using the personal bag frequently or every day, while two participants reported rarely using their personal bags. No alleles were detected on zip lock bags after storage in rarely used personal bags. 

### 3.2. Persistence and Detection of DNA from a Previous User of a Tape Roll Used to Wrap Drugs

**Figure 3:**
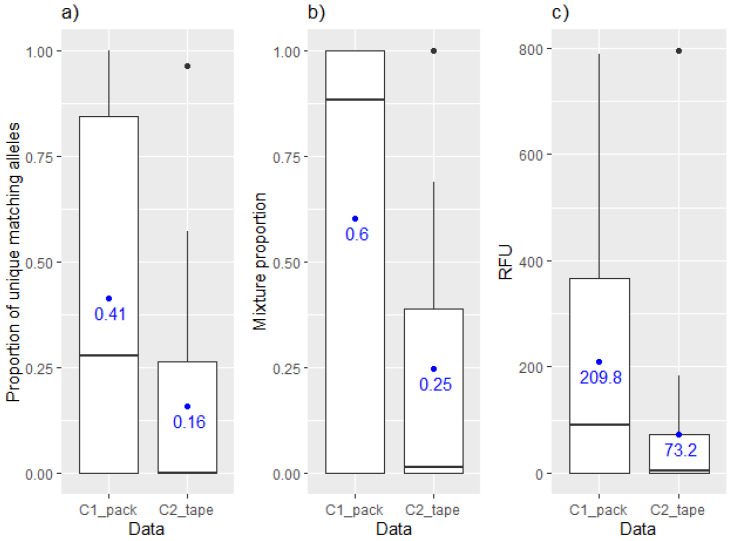
Boxplot displaying (**a**) proportion of unique alleles, (**b**) the mixture proportion and (**c**) $\bar{RFU}$ values corresponding to the wrapper (C1_pack_) and the handler (C2_tape_) of the tape.

In samples collected from wrapped cardboard, DNA from both the person who previously handled the tape (C2_tape_) and the person who packed the tape around the cardboard (C1_pack_) could be detected. However, the quality of the profile, peak heights of detected alleles and mixture proportions varied between the cardboard wrapper and the tape handler (Figure 3).

Three samples had no profiling results and for an additional three samples only four alleles were detected. Seven samples were full or partial profiles corresponding to the drugs wrapper (C1), five samples were mixtures of C1 and C2. For three of these mixtures, C1 was the major contributor, whilst in the last two, the contributions were of equal proportions. Two samples were partial profiles with alleles corresponding to C2. The full dataset can be found in the Supplementary Material S2.

### 3.3. Shedder Status

**Figure 4:**
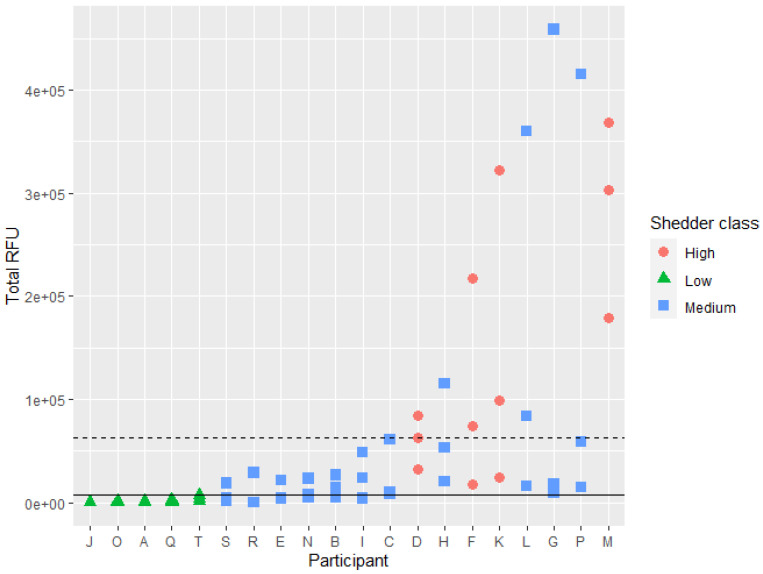
Scatter plot showing the total *RFU_tot_* values (multiplied by *M_x_* if sample was a mixture) for each sample collected from each participant and according to their shedder status, high (red circle), medium (blue square) low (green triangle). The average *RFU_tot_* (6.25E+04) and low shedder limit of 8.00E+03 *RFU_tot_* are represented with the dotted and straight lines, respectively.

The total *RFU* values (*RFU_tot_*) from the direct PCR analysis varied from 0 to 458,705, variation was greater between individuals than within the samples from one individual (ANOVA, *p* = 0.04), Figure 4. However, some individuals, especially those that deposited low amounts of DNA, displayed lower variation. Individuals were classified as low, medium or high shedders based on the criteria defined by Johannessen et al. [15] with the following adjustments according to the observed values of the current dataset: high shedder class was assigned to participants that had two out of three samples above the average *RFU_tot_* (62,520) and two out of three samples with 20 out of 24 full loci. Low shedder class was assigned to participants where all samples were below 8000 *RFU_tot_* and two out of three with negative or partial profiles. The rest were classified as medium shedders. This resulted in 4 high, 11 medium and 5 low shedders (Figure 3). The full dataset can be found in the Supplementary Material S2.

DNA from unknown contributors was detected in 30/90 samples, the average *M_x_* proportion from unknown contributors was highest for low shedders, Table 1. 

### 3.4. Transfer in Relation to Shedder Status

**Figure 5:**
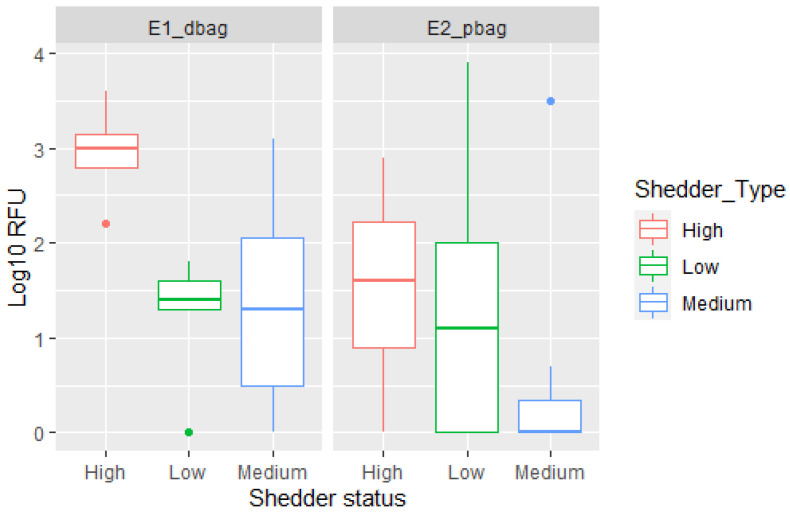
Boxplot displaying the log10 $\bar{RFU}$ detected in the direct transfer experiment ($E1_{dbag}$) and indirect transfer from personal bag ($E2_{pbag}$) according to the participants’ shedder classification.

Data from experiment 1 (direct transfer to zip-lock drugs bag) showed highest transfer rates with high shedders (Figure 5). More variation was observed in the medium shedder group, than for the low and high shedders. The trend is not as obvious for experiment 2 (indirect transfer from personal bag to zip-lock drugs bag). In fact, the best quality profile was detected in a sample from a low shedder participant. Based on the number of samples in each group, there were insufficient data to evaluate the effect using Bayesian networks described in Section 3.5.1.

**Figure 6:**
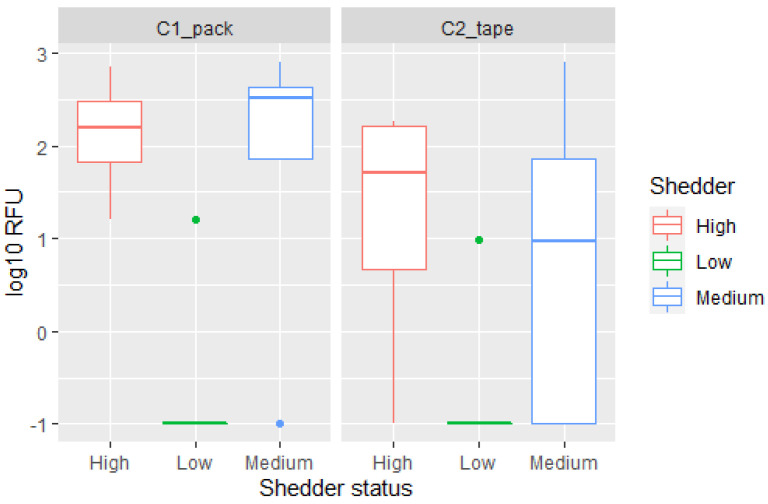
Boxplot displaying the log10 $\bar{RFU}$ for the drugs wrapper (C1_pack_) and the previous handler (C2_tape_) of the tape according to the shedder classification of the participants.

Data from persistence and detection of DNA from a previous user of a tape roll experiment showed that low shedders only provided low quality profiles and that higher quality profiles were provided by high shedders. Medium shedders provided both high and low quality results. (Figure 6).

### 3.5. Case Examples

To demonstrate the potential use of the datasets obtained, we will use two fictive case examples that are based on actual experiences from case work, using a Bayesian network to evaluate the evidence. 

#### 3.5.1. Potential DNA Transfer from Storage in a Personal Bag

##### Case Circumstances

1. A large depot of drugs was found at a hideout;
2. The drugs were packed in zip-lock bags and placed in a black gym bag;
3. Upon questioning, person A claims to have no knowledge of the drugs. However, he recognizes a gym bag that the drugs had been stored in and claims that it used to belong to him but it was lost or stolen two weeks previously.

##### DNA Analysis

DNA samples were collected from the outside and opening of the zip-lock bag

4. Example 1: Result is a full DNA profile of a single individual. There is a candidate in the national DNA database who is identified as person A;
5. Example 2: Result is a mixture of two individuals. There is a candidate in the national DNA database who is identified as person A but there is no candidate for the second individual.

##### Propositions

Sub-source likelihood ratios were calculated for both contributors using EuroForMix [21], although this information was not used in any further analysis. To proceed with activity level analysis, it is assumed that the sub-source LR is sufficient for a court to agree the identity of a POI [2].

The alternative activity level propositions (for both examples) are as follows:

*H_p_*: The suspect packed the drugs;

*H_d_*: The suspect has no relation to the drugs but was the owner of the gym bag which was stolen two weeks previously. An unknown individual packed the drugs.

**Figure 7:**
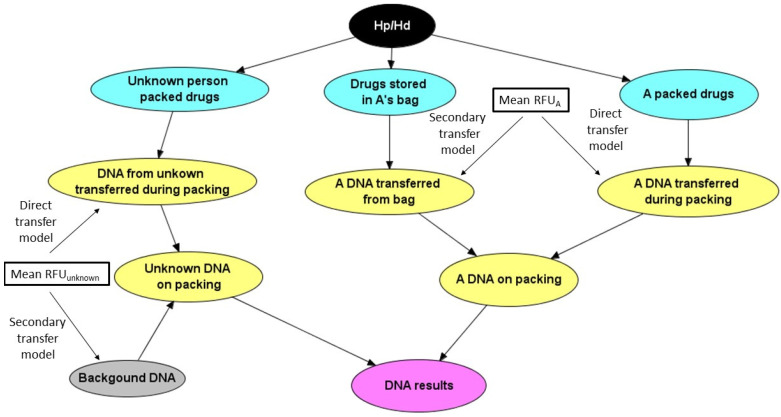
Bayesian network for the “drugs stored in gym bag” example showing data sets and models used to instantiate the nodes.

**Figure 8:**
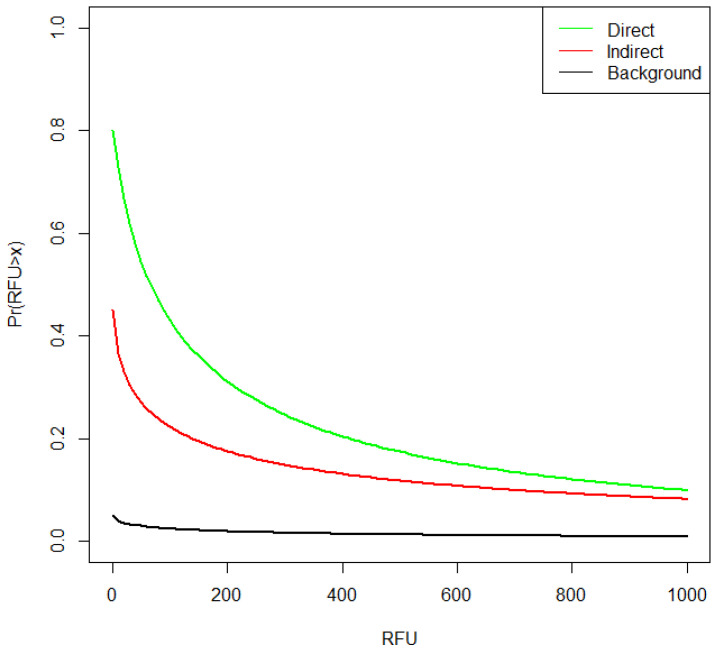
Comparison of probabilities of direct, indirect and background TPPR for the zip-lock drugs bag example. Direct transfer was modelled from ${\bar{RFU}}_{E1dbag}$; personal bag (indirect transfer) was modelled from ${\bar{RFU}}_{E2pbag}\;$ data and background was also modelled from ${\bar{RFU}}_{E2pbag}$ data, with adjusted *k* = 0.95 (the proportion of observations with no background) in order to scale results as described in Supplementary Material S1.

The BN used to instantiate probabilities is identical to that described by [6] (Supplement S1), except that the title of the nodes are changed to represent the case circumstances (Figure 7). Log-normal distributions were used to calculate drug bag (direct) and personal bag (indirect) TPR probabilities, conditioned upon values of ${\bar{RFU}}_{A}$ modelled with ${\bar{RFU}}_{E1dbag}$ and ${\bar{RFU}}_{E2pbag\;}$ distributions respectively. ${\bar{RFU}}_{unknown}$ was modelled with ${\bar{RFU}}_{E2pbag}$ distribution (Table 2), the assigned probabilities (Figure 8) were subsequently used in the BN (Figure 7) to calculate the results. To carry out sensitivity analysis, 1000× bootstraps (with replacement) were taken of the $\bar{RFU}$*_E1_dbag_* and $\bar{RFU}$*_E2_pbag_* data (Supplementary Material S2) to provide 1000 new sets of data. For each bootstrap, a new set of log-normal parameters (mean log and SD log) were calculated using the *fitdistrplus* R package and the variation was represented by percentiles in Table 3.

Derivation of the formulae used in the calculations are the same as those described in Supplementary Material S1 of Gill et al. [6]. Likelihood ratios were calculated as shown in Formulae 3–5 (nomenclature in Table 2):

(a) Only the POI (A) is observed
  (3) $$LR=\frac{\left(s\left(1-t_{A}\right)+t_{A}\right)\times \left(1-b\right)}{s\times \left(1-\mathrm{Pr}\left(U_{d}\right)\right)}$$
(b) POI (A) and unknown is observed
  (4) $$LR_{a}=\frac{\left(s\left(1-t_{A}\right)+t_{A}\right)\times b}{s\times \mathrm{Pr}\left(U_{d}\right)}$$
(c) Probability of recovery of DNA from an unknown contributor
  (5) $$\mathrm{Pr}\left(U_{d}\right)=t^{\prime }b+t^{\prime }\left(1-b\right)+b\left(1-t^{\prime }\right)=t^{\prime }+b\left(1-t^{\prime }\right)$$

There were only three observations of background (datasets $E1_{dbag}$ and $E2_{pbag}$). Background is from indeterminate (unknown) sources and can comprise both direct and indirect transfer. Here, it was modelled using ${\bar{RFU}}_{E1pbag}>x$ data (where *x* is a threshold value; first column in Table 3), except that the model was scaled relative to *k* = 1 − Pr(*b*) as described in the Supplementary Material S1; where *k* is the proportion of observations where no background was observed. From experimental observation Pr(*b*) = 0.05 and *k* = 0.95.

Two possible outcomes of the DNA results were analysed in detail. Either a profile from the POI individual A is recovered, or A is recovered in combination with an unknown contributor which forms the basis of the proposition under *H_d_*, else the unknown contributor is background under *H_p_*.

##### Contributor A Recovered Alone

When contributor A is recovered alone, Table 3 shows that the median (50 percentile) LRs always favour the proposition that he/she packed the drugs; the evidence provides moderate support. If a discrete model is used where allele peak height is not considered, then LR = 11 (top row of Table 3). Taking peak height into account has little effect in this example. The sensitivity analysis shows the evidence always favours *H_p_* at the 2.5 percentile.

##### Unknown and Contributor A Recovered

A different result is obtained when a mixture of unknown and contributor A is recovered. For the discrete model (top row of Table 4) the LR ≈ 0.1, favouring the proposition that an unknown contributor wrapped the drugs. When allele peak height is considered, similar results are obtained (Table 4).

#### 3.5.2. Cardboard Drug Wrap Experiment

##### Case Circumstances

A large depot of drugs was detected by the police during a house search of A’s house.

The drugs were wrapped in cardboard and covered by packing tape; 

Person A admits that he packed the drugs and does not implicate anyone else;

Person B claims to have no knowledge of the drugs. However, he worked for a moving agency and often handles packing tape. Remains of packing tapes are often left behind and could be picked up and used by others. 

##### DNA Analysis

DNA samples were collected from the wrappings. The results of the DNA analysis were a mixture of person A and an unknown contributor. A candidate was identified as person B from a national DNA database search.

##### Propositions

Sub-source likelihood ratios were calculated for both contributors using EuroForMix [20], although this information was not used in any further analysis. To proceed with activity level analysis, it is assumed that the sub-source LR is sufficient for a court to agree the identity of a POI [2].

The prosecution and defence activity level propositions are as follows:

*H_p_*: A and B packed the drugs together and B did not previously handle the tape;

*H_d_*: A packed the drugs alone; B had previously handled the tape

**Figure 9:**
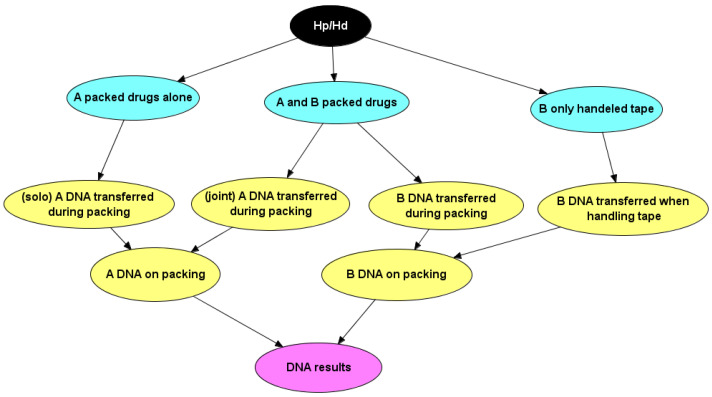
Bayesian network for the evaluation of evidence in the case where DNA evidence was collected from cardboard drug wrap.

From the case information, a Bayesian network incorporating the relevant nodes was prepared (Figure 9). See Supplementary Materials S1 for details. Since both *H_p_* and *H_d_* agree that contributor A packed the drugs, his/her presence or absence of DNA has no effect upon the likelihood ratio. Only contributor B has an effect.

##### Statistical Analysis

**Figure 10:**
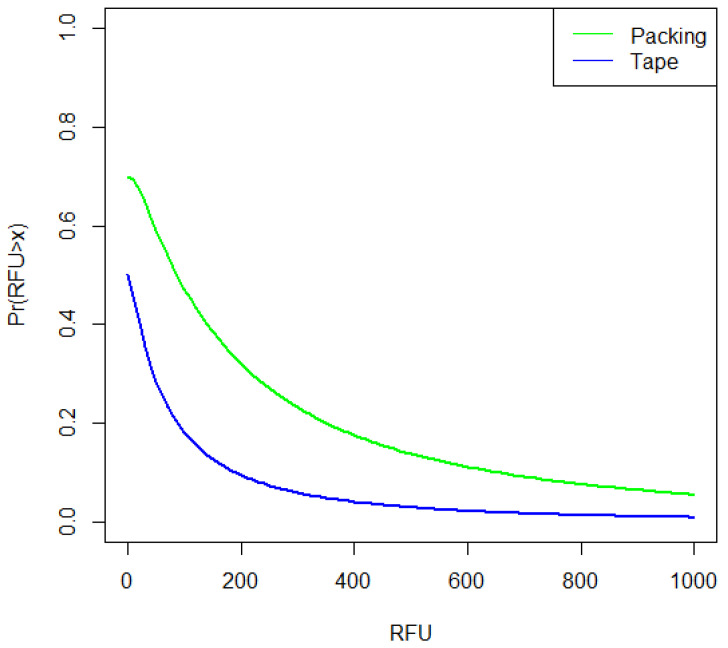
Lognormal distributions of ${\bar{RFU}}_{C1pack}\;\mathrm{and}\;{\bar{RFU}}_{C2tape}$.

The data are provided in Supplementary Materials S2. In the experiment, contributor B previously handled the tape as described in Section 3.5.1, and contributor A packed the drugs. The mean $\bar{RFU}$ of the observed DNA profile was split per contributor (${\bar{RFU}}_{A}$ and ${\bar{RFU}}_{B}$), based upon their respective mixture proportions (*M_x_*): i.e., ${\bar{RFU}}_{A}={\bar{RFU}}_{tot}\times M_{xA}$ and ${\bar{RFU}}_{B}={\bar{RFU}}_{tot}\times M_{xB}$, where *M_x_* was calculated using EuroForMix [20]. Log-normal distributions were fitted to each set of data using the *fitdistrplus* R package (Figure 10). Distributions for the BN node “B DNA transferred when handling tape” (Figure 9) were modelled from ${\bar{RFU}}_{C2tape}$; distributions for BN nodes” (solo) A DNA transferred during packing” and “(joint) B DNA transferred during the packing” were both modelled from ${\bar{RFU}}_{C1pack}$ (Table 5).

Likelihood ratios were calculated as follows (see Table 5 for context):

(a) A DNA is recovered: $LR=\frac{\left(1-t_{B}\right)}{\left(1-s\right)}$
(b) B DNA is recovered: $LR=t_{B}/s$
(c) A and B DNA are recovered: $LR=t_{B}/s$
(d) no DNA is recovered: $LR=\frac{\left(1-t_{B}\right)}{\left(1-s\right)}$

The formulae are derived in Supplementary Material S1. Note that likelihood ratios of (a) and (d) are the same, as are (b) and (c). This is because both *H_p_* and *H_d_* condition upon contributor A, hence his/her presence is cancelled out. To carry out sensitivity analysis, 1000× bootstraps (with replacement) were taken of the ${\bar{RFU}}_{A}$ and ${\bar{RFU}}_{B}$ data (Supplementary Material S1). For each bootstrap, a new set of log-normal parameters (mean log and SD log) were calculated using the *fitdistrplus* R package.

##### Results of Analysis

Log-normal distributions were used to calculate the tape handling and drug packing TPR probabilities, conditioned upon values of ${\bar{RFU}}_{B}>x$ from simulated profiles and subsequently used in the BN (Figure 8) to calculate the results (Table 6). 

Likelihood ratios, along with sensitivity analyses are calculated against a range of $RFU_{B}>x$ values (Table 6). If a profile is obtained where only contributor A is present and there is no DNA present that can be attributed to contributor B, then the LR = 0.6, i.e., supports the proposition that suspect B did not package the drugs. If DNA is present that can be attributed to B, then the LR > 1, favouring the proposition that suspect B did package the drugs. The presence/absence of individual B as a contributor to the evidence can be described either as discrete: Pr(${\bar{RFU}}_{B}>0$) vs. absence, or as a continuous distribution where Pr(${\bar{RFU}}_{B}>x$), where x is a threshold value. With a discrete model, LR=1.4 (first row of Table 6). Taking the value of ${\bar{RFU}}_{B}>x$, with the continuous model in subsequent rows of Table 6, a higher LR is achieved, reaching a maximum median LR=6.9 if RFU_B_ > 1000 (the limit of observations in Supplementary Materials S2), although the evidence can only be described as “weak” following the ENFSI verbal scale [22]. The sensitivity analysis shows the observed range between 1–99 percentiles from 1000× bootstraps of the data. The variation increases greatly as ${\bar{RFU}}_{B}$ increases—a reflection of the small size of the datasets. In conclusion, the discrete model understates the value of the evidence, compared with the continuous model.

## 4. Discussion

### 4.1. Zip-Lock Drugs Bag Experiment

The DNA from the POI was detected more frequently on a zip-lock drugs bag if the bag was directly handled. However, the best quality profile observed in the study was collected from a zip-lock drugs bag stored in a personal bag (purse). From the literature, indirect transfer to a sleek surface such as the zip-lock drugs bag is expected to be low [8]. Most of the personal bags used in the experiments had previously been frequently used by the participants, although there are few observations in each class of bags, there is an indication that larger quantities of DNA can be detected on a zip-lock drugs bag after storage in purses. More DNA could be accumulated from the user on the inside of a purse as several personal items (e.g., phone, hairbrush, wallet) containing owner DNA are frequently stored there. In addition, the surface of the inside of the bag will influence accumulation and further transfer [8,23]. In the case example, it is shown that personal bags that are used to store everyday items, such as clothing or personal effects that are frequently handled, accumulate amounts of DNA that may be indirectly transferred to other objects. This resulted in low likelihood ratios (moderate evidence to support *H_p_*) when DNA from only the POI was recovered. There was little difference between the discrete model and the continuous model, the latter takes the allele peak height into account. With mixtures, both discrete and continuous models favour *H_d_* (LR ≈ 0.1; Table 4). In the experiment, only low amounts of background (DNA from unknown contributors) were detected. Combined with the relatively low difference between indirect and direct transfer probabilities (Figure 8), especially when $\bar{RFU}>1000$, this has an impact of reducing the LR if the sample is in admixture with an unknown contributor, so that it always favours the defence proposition (*H_d_*). 

### 4.2. Cardboard Drug Wrap Experiment

In this study, the persistence of DNA from previous handlers of a roll of tape was investigated. As the top surface of the tape is hard and non-porous, the expectation is that low amounts of DNA would be transferred and detected after direct contact, in addition to a rapid removal of DNA from the previous user [8,12,13]. The findings of this study generally correlate with these expectations: a large proportion of samples produced no results or only a few alleles. The packer (last handler) was the only contributor or the one contributing a larger amount of DNA in most of the samples that gave results. Some exceptions were observed with two profiles showing results that only corresponded to the tape handler (person B). The sides of the tape have a rougher surface where more DNA is expected to be transferred upon initial contact [9,23]. In addition to DNA from the previous user persisting on the surface of the tape, it is possible that some of the DNA deposited on the side of tape could be transferred to the new user’s hands and to the cardboard during the wrapping of the drugs. As no other items were touched in between handling the tape and performing the wrapping, there was no opportunity for the loss of person B’s DNA to other surfaces. We did not monitor the wrapping procedure and recognise that the manner of contact during the procedure could influence the result. However, information regarding this will rarely be known in casework. 

In the Bayesian network case example, we considered that an individual B claims that he/she handled tape which was later used to prepare drug wraps. Individual A has admitted the offence, hence, his/her presence of DNA on the drug wrap has no bearing on the value of the evidence. If contributor B’s DNA is recovered, without taking account of $\bar{RFU}$ in the discrete model, the evidence is close to neutral LR = 1.4, whereas if $\bar{RFU}$ is taken into account, the value of the evidence increases with RFU, although it does not exceed LR = 7 where $\bar{RFU}>1000$ (the upper limit of experimental observations), i.e., the evidence would be described as weak using the ENFSI scale [22]. However, if B’s DNA is absent, then this favours the defence proposition LR = 0.6. 

The experimental set up is similar to that used in [13] where the question was if the POI previously cut an aluminium foil or used this foil in lock picking. The activity LR in the lock-picking study, calculated with a discrete model (7.4) was greater than that observed in the current study (1.4). However, the LR was in the same range as the maximum (median) level of the continuous model employed (LR = 6.9). Some of the differences could be explained by the difference in transfer to and persistence on aluminium foil vs. tape. 

### 4.3. Shedder Status and Transfer Probabilities

**Figure 11:**
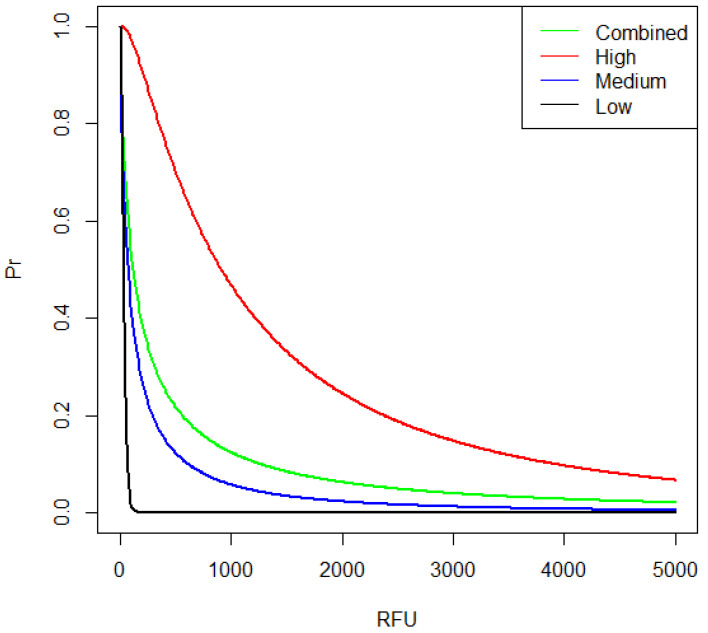
Effect of high/medium/low shedder status on direct transfer (E1) data.

A person’s shedder status has previously been shown to influence the probability of transfer, persistence, and detection of DNA [14,24]. Fonneløp et al. [14] demonstrated that DNA was more frequently detected in samples collected from the T-shirts of a victim if the attacker was a high shedder and that the probability of detection increased further if the victim was a low shedder. While Otten et al. [24] observed a correspondence between shedder stratus and DNA transfer to gloves. The correspondence between shedder status and the amount of DNA transferred is further demonstrated by our findings when it comes to direct transfer to zip-lock drugs bags (Figure 11), direct transfer during wrapping with a tape and transfer and persistence after touching a role of tape. On the other hand, when indirect transfer from the inside of a personal bag was considered, no clear association with shedder status was observed, and the best quality profile was detected after storage in a low shedder’s personal bag. We hypothesised the amount of previous use and the surface of the inside of the bag could be of higher importance for this type of transfer. The low number of samples collected in each category is also a limitation and a clearer correspondence may be seen with a larger collection of data. Shedder status was not incorporated into the Bayesian networks because dividing the results into three categories would lead to too few data in each group to analyse. Secondly, it would also be important to properly characterise the effect of shedder status on indirect transfer before applying this variable to the model.

The LRs calculated in this study are comparable to other studies where DNA transferred by hands is considered [6,13,25]. It is likely that including more information—especially shedder status, would, to some degree, change the LR calculations [14]. 

### 4.4. Detection of Unknown DNA

This experiment was performed during the COVID-19 pandemic where a general recommendation to keep a social distance of at least two meters and to wash hands frequently or use antibacterial liquid was given. It is likely that these measures could have had an impact on the detection of unknown DNA in the samples, which was low compared to previous studies [9,16,26]. 

## 5. Conclusions

We have created datasets on direct and indirect transfer to zip-lock bags and transfer and persistence to tape and further shown how the data can be used to inform Bayesian Networks. As the indirect and persistence scenarios tested are realistic under the circumstances utilised in this study, only moderate to low support for *H_p_* was obtained. We have shown that applying a continuous model based on the profile quality can alter LRs compared to a discrete model and is preferable. There are challenges with limited datasets, and we were not able to implement shedder status into our models. More data on the influence of shedder status are required when indirect transfer is considered. 

## Figures and Tables

**Table 1:** Detection of unknown alleles in the shedder samples and the proportion of unknown DNA detected for the three different shedder classes.

	Number of Samples	Average *M_x_* Unknown Contrib.
High	4/12 (33%)	7.1
Medium	19/33 (58%)	8.5
Low	7/15 (47%)	52.0

**Table 2:** Relationship of probabilities of DNA transfer from individuals A (POI) and U (unknown), showing the datasets that were used to calculate probability distributions, along with the BN nodes instantiated. See nomenclature Section 2.5.1 for definitions of probabilities.

Probability	Description	Contributor	Data used to Inform Probability Distribution	BN Nodes
*t_A_*	Packing transfer	A	RFU_E1dbag_	A DNA transferred during packing
*t’*	Packing transfer	U	RFU_E1dbag_	DNA from unknown transferred during packing
*s*	Bag transfer	A	RFU_E2pbag_	A DNA transferred from bag
*b*	Background	U	RFU_E2pbag_	Background DNA

**Table 3:** Activity level likelihood ratios where only contributor A is recovered, with sensitivity analysis, showing 2.5–97.5 percentiles. The median (50 percentile) values are those that are reported. Results for the discrete model are shown in the top row; continuous models conditioned upon ${\bar{RFU}}_{A}>x$ are shown in remaining rows.

RFU_A_ > x	2.50%	5%	10%	25%	50%	75%	90%	95%	97.50%
0	5	5	6	8	11	17	27	39	6 × 10^1^
200	5	5	7	9	14	23	47	82	2 × 10^3^
400	4	5	6	9	13	24	52	122	1 × 10^4^
600	4	5	6	8	12	24	52	153	4 × 10^4^
800	4	5	6	8	12	24	53	196	9 × 10^4^
1000	4	4	5	8	12	24	54	218	2 × 10^5^
2000	4	4	5	7	11	24	63	448	3 × 10^6^
4000	3	4	4	6	10	23	77	1016	6 × 10^7^
6000	3	4	4	6	9	21	87	1752	4 × 10^8^

**Table 4:** Activity level likelihood ratios where contributor A and an unknown are recovered, with sensitivity analysis, showing 2.5–97.5 percentiles. The median (50 percentile) values are those that are reported. Results for the discrete model are shown in the top row; continuous models conditioned upon ${\bar{RFU}}_{A}>x$ and ${\bar{RFU}}_{unknown}>x$ are shown in remaining rows.

RFU_A_ > x	RFU_Unknown_ > x	2.50%	5%	10%	25%	50%	75%	90%	95%	97.50%
0	0	0.1	0.1	0.1	0.1	0.1	0.1	0.2	0.2	0.2
100	2000	0.1	0.1	0.1	0.2	0.3	0.6	1.1	1.5	2
100	7000	0.1	0.2	0.2	0.3	0.6	1.1	1.8	2.4	4
2000	100	0.1	0.1	0.1	0.1	0.1	0.2	0.5	1.4	24
7000	100	0.1	0.1	0.1	0.1	0.1	0.1	0.6	3.6	307
1000	2000	0.1	0.1	0.2	0.2	0.3	0.5	0.9	1.5	20
1000	7000	0.2	0.2	0.2	0.3	0.5	0.9	1.3	2.7	35
2000	1000	0.1	0.1	0.1	0.1	0.2	0.3	0.7	1.8	45
7000	1000	0.1	0.1	0.1	0.1	0.2	0.3	1.0	4.2	717

**Table 5:** Relationship of probabilities of DNA transfer from individuals A and B, showing the datasets that were used to calculate probability distributions, along with the BN nodes instantiated. Nomenclature is described in Section 2.5.1.

Probability	Description	Contributor	Data Used to Inform Probability Distribution	BN Nodes
*t_A_*	Packing transfer	A	RFU_C1pack_	(solo) A DNA transferred during packing (joint) A DNA transferred during packing
*t_B_*	Packing transfer	B	RFU_C1pack_	(joint) B DNA transferred during packing
*s*	Tape transfer	B	RFU_C2tape_	B DNA transferred when handling tape

**Table 6:** Activity level likelihood ratios, with sensitivity analysis, showing 1–99 percentiles. The median (50 percentile) values are those that are reported. Results for the discrete model are shown in the top row; continuous model conditioned upon ${\bar{RFU}}_{B}>x$ shown in remaining rows.

RFU_B_ > x	1%	2.50%	5%	10%	25%	50%	75%	90%	95%	97.50%	99%
0	0.8	0.8	0.9	1.0	1.2	1.4	1.7	2.0	2.3	2.6	3.0
100	1.2	1.4	1.5	1.7	2.1	2.7	3.9	5.6	7.3	10.0	16.2
200	1.3	1.5	1.7	2.0	2.6	3.7	6.6	12.9	20.5	34.0	90.5
300	1.3	1.5	1.7	2.1	2.8	4.5	10	23	43	93	282
400	1.3	1.5	1.7	2.0	3.0	5.1	13	39	78	190	953
500	1.2	1.4	1.6	2.0	3.1	5.5	17	65	147	371	2152
600	1.1	1.3	1.5	1.9	3.0	5.9	20	97	238	603	4441
700	1.0	1.2	1.4	1.8	3.0	6.1	24	133	360	1157	8454
800	0.8	1.1	1.3	1.7	3.0	6.4	29	179	563	1872	15,118
900	0.7	1.0	1.3	1.6	3.0	6.7	35	227	833	2951	27,012
1000	0.6	1.0	1.2	1.6	2.9	6.9	40	291	1176	5202	52,019

## Data Availability

The data supporting the findings reported in this manuscript can be found in the supplementary material S2, Tables S1–S3.

## Supplementary Materials

The following are available online at https://www.mdpi.com/article/10.3390/genes13010018/s1, Supplementary Material S1: Derivation of formulae from experiment 2: Cardboard drug wraps, Supplementary Material S2: Supplementary table 2.1 Results from the direct and indirect transfer to zip-lock bags experiment; Supplementary table 2.2 Results from the persistence and detection of DNA from a previous user of a tape roll experiment; Supplementary table 2.3 The results of the shedder status experiment.
