# Supplementary material for: Who Packed the Drugs? Application of Bayesian Networks to Address Questions of DNA Transfer, Persistence, and Recovery from Plastic Bags and Tape
Source: Genes (Basel). 2021 Dec 22;13(1):18. doi: 10.3390/genes13010018 (PMC8774669; doi:10.3390/genes13010018)
Supplement: Supplementary file 1 [file genes-13-00018-s001.zip › Supplementary material/Supplementary material S1.pdf]

Supplementary material S1

Derivation of formulae from experiment 2: Cardboard drug wraps

1. **Case circumstances**

Both H_p and H_d agree that Mr. A packaged the drugs. H_p asserts that Mr B packaged the drugs along with Mr. A. H_d asserts that he did not package the drugs, he had previously handled the tape that was subsequently used by Mr. A. The proposition under H_p discounts this possibility.

There are four possible outcomes in the results (discounting mixtures from unknown contributors with this example)

Only A is observed

Only B is observed

A and B are observed

No DNA is observed

2. **Nomenclature:**

t_A: Direct transfer from Mr. A

t_B: Direct transfer from Mr. B

s: Transfer from Mr B previously handling the tape

3. **Outcomes**

3.1 Only A is observed

Under *Hp:*

DNA has transferred from Mr A because of packing the drugs jointly with Mr. B. DNA from Mr B has not transferred during packing. The probability of this is $t_A(1 - t_B)$

Under *Hd*:

the DNA from A has come from packing and DNA has not transferred from previous contact with the tape by *B* The probability of this is $t_A(1 - s)$

The likelihood ratio is: $LR = \frac{(1-t_B)}{(1-s)}$

3.2 Only B is observed

Under H_p:

Mr B handled the tape with Mr. A . DNA from A was not transferred during the packing, but DNA from B was. The probability of this is $t_B(1 - t_A)$

Under H_d:

Mr B only handled the tape. Mr A's DNA was not transferred during packing. This probability is $s(1 - t_A)$

The likelihood ratio is: $LR = \frac{t_B(1-t_A)}{s(1-t_A)} = \frac{t_B}{s}$

3.3 A and B are observed

Under H_p:

Mr B handled the tape with Mr. A The probability of this is $t_A t_B$

Under H_d:

DNA has transferred from Mr A during packing and DNA has transferred from Mr B to the package as a result of him previously handling the tape. The probability of this is st_A

The likelihood ratio is $LR = \frac{t_B}{s}$

3.4 No DNA observed

Under H_p,

No DNA has been transferred from Mr A or Mr B during packaging. The probability is $(1 - t_A)(1 - t_B)$

Under H_d,

DNA has been transferred from Mr A during packaging or from Mr B from previously handling tape. The probability is $(1 - t_A)(1 - s)$

The likelihood ratio is $LR = \frac{1-t_B}{1-s}$

4. **Log-normal Distribution fitting to the data**

The analysis of the data files in supplement 2 were carried out with the R package *fitdistrplus* using the *lnorm* function to fit log-normal distributions of $\overline{RFU}$, the mean RFU of a profile, possibly scaled with mix-prop. to obtain individual specific contribution. Data where $\overline{RFU} < 0.1$ were excluded from the preliminary analysis, because there are a large number of values at zero (or close to zero) which cannot be modelled. The *plnorm* function computes the cumulative probability Pr($\overline{RFU}' < x$), where *x* is a threshold $\overline{RFU}'$ value; note $\overline{RFU}'$ signifies that only non-zero $\overline{RFU}$ data are included in this analysis.

$$Pr(\overline{RFU}' < x) = \Phi((lnx - \mu)/\sigma)$$

Φ is the cumulative density function of the normal distribution and μ and σ are fitted model parameters.

Probabilities must be assigned from the complete dataset, hence rescaling is required to include the proportion of data (*k*) where $\overline{RFU} < 0.1$. Also we want to determine the probability that $\overline{RFU}$ is *greater* than threshold value *x*. The rescaled value is:

$$Pr(\overline{RFU} > x) = 1 - (k + ((1 - k) \times Pr(\overline{RFU}' < x))$$

Provided that x>0.

4.1. Case example 1: The zip-lock drugs bag experiment data

4.1.1. Analysis of $\overline{RFU}$ dataset from personal bag data $E2_{pbag}$

The probability of secondary transfer (*s*) is assigned from log normal distributions fitted to mean $\overline{RFU}_{E2pbag}$ data - the personal bag data from supplement 2 (fig. S1). The fitted parameters were mean log = 4.58 and sd log = 2.57. The secondary transfer probabilities were derived from E2 data, the same as described in the previous section. Background was rarely observed in this dataset and was assigned a value Pr(*b*)=0.05. Since there were too few observations to model, it was assumed to have the same distribution of dataset $\overline{RFU}_{E2pbag}$; rescaled to *k*=0.95 (as described in section 4, this supplement).

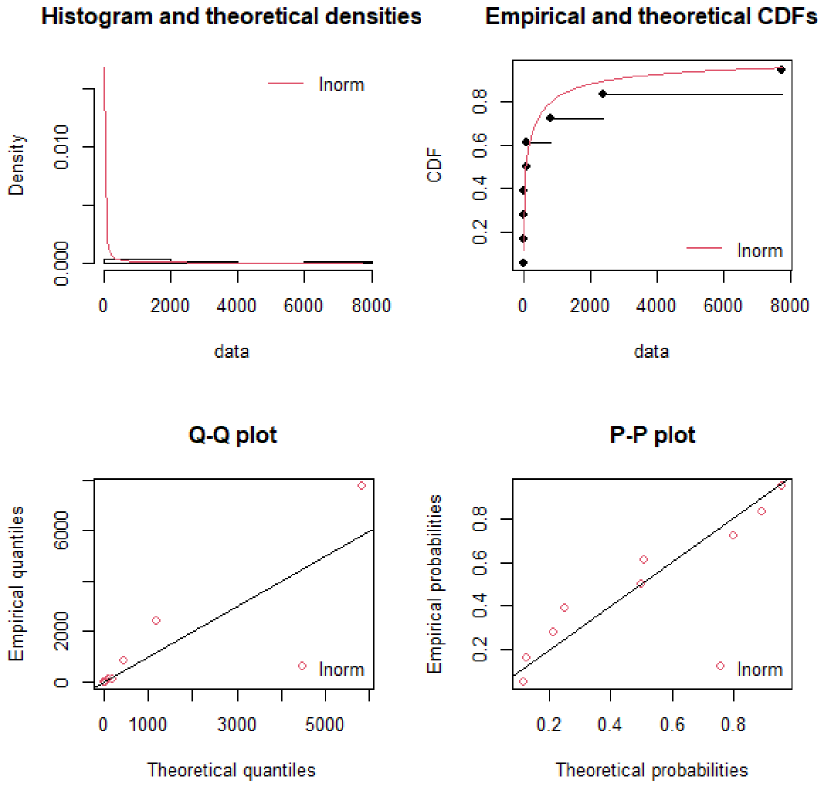

Figure S1: Log normal distribution fit to the personal bag data $\overline{RFU}_{E2pbag}$.

4.1.2. Analysis of $\overline{RFU}$ dataset from ziplock drugs bag data $E1_{dbag}$

Log normal distribution fitted to $\overline{RFU}_{E1dbag}$ data (direct transfer to the ziplock drugs bag) provided parameters: mean log=4.78 and sd log= 1.84 (fig S2)

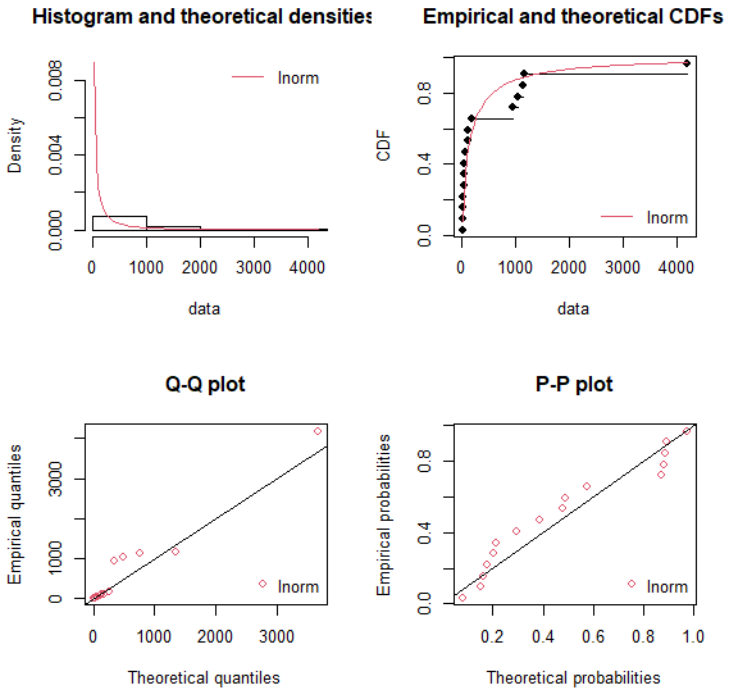

Figure S2: Log normal distribution fit to the ziplock drugs bag data $\overline{RFU}_{E1dbag}$.

4.2 **Case example 2: The cardboard drug wraps**

4.2.1. Analysis of $\overline{RFU}$ dataset from drugs packer ($C1_{pack}$).

Log normal distribution fitted to $\overline{RFU}_{C1pack}$ data (direct transfer to the ziplock drugs bag) provided parameters: mean log=5.16 and sd log = 1.24 (fig S3).

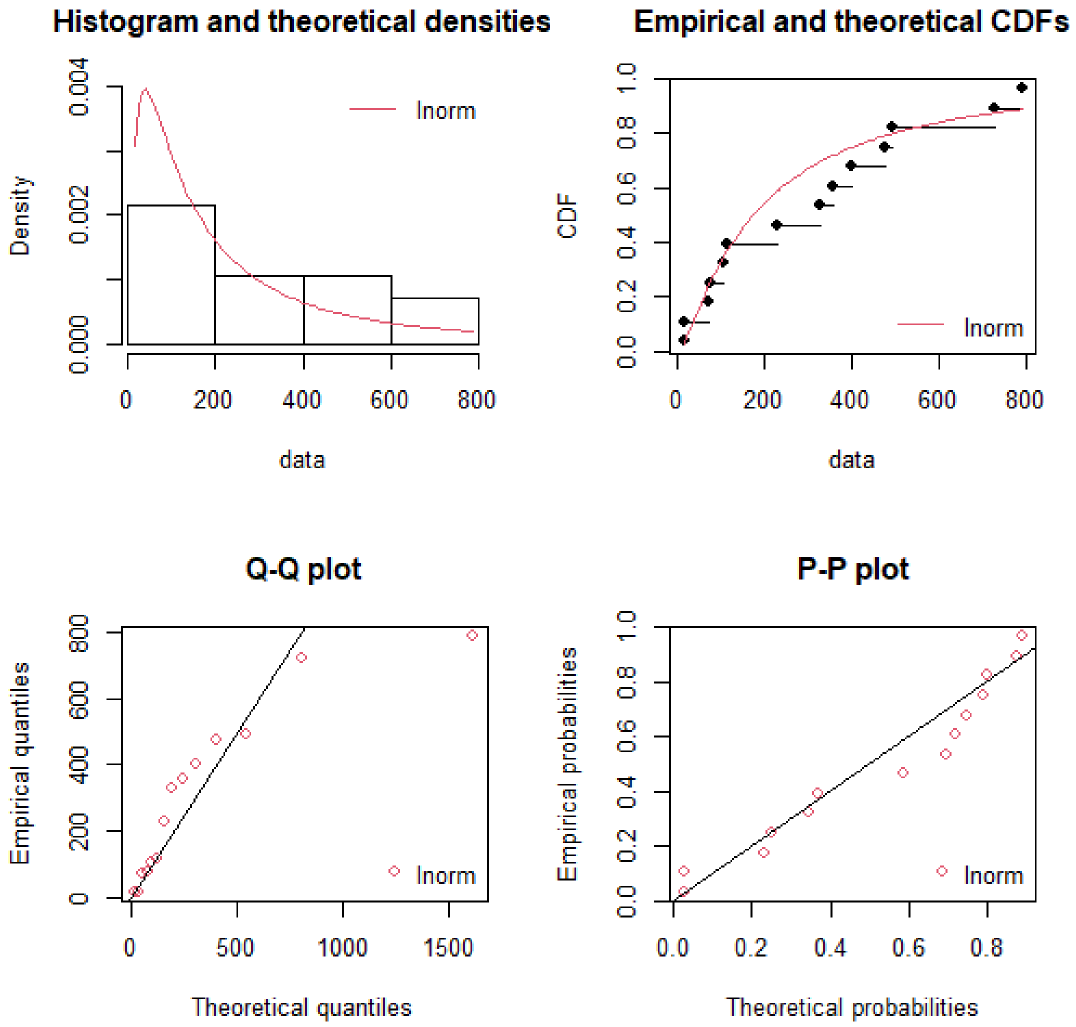

Figure S3: Log normal distribution fit to the ziplock drugs bag data $\overline{RFU}_{C1pack}$.

4.2.2. Analysis of $\overline{RFU}$ dataset from previous user of tape (*C2$_{tape}$*).

Log normal distribution fitted to $\overline{RFU}_{C2tape}$ data (direct transfer by a previous user of the tape) provided parameters: mean log = 4.14 and sd log = 1.32. (fig S4).

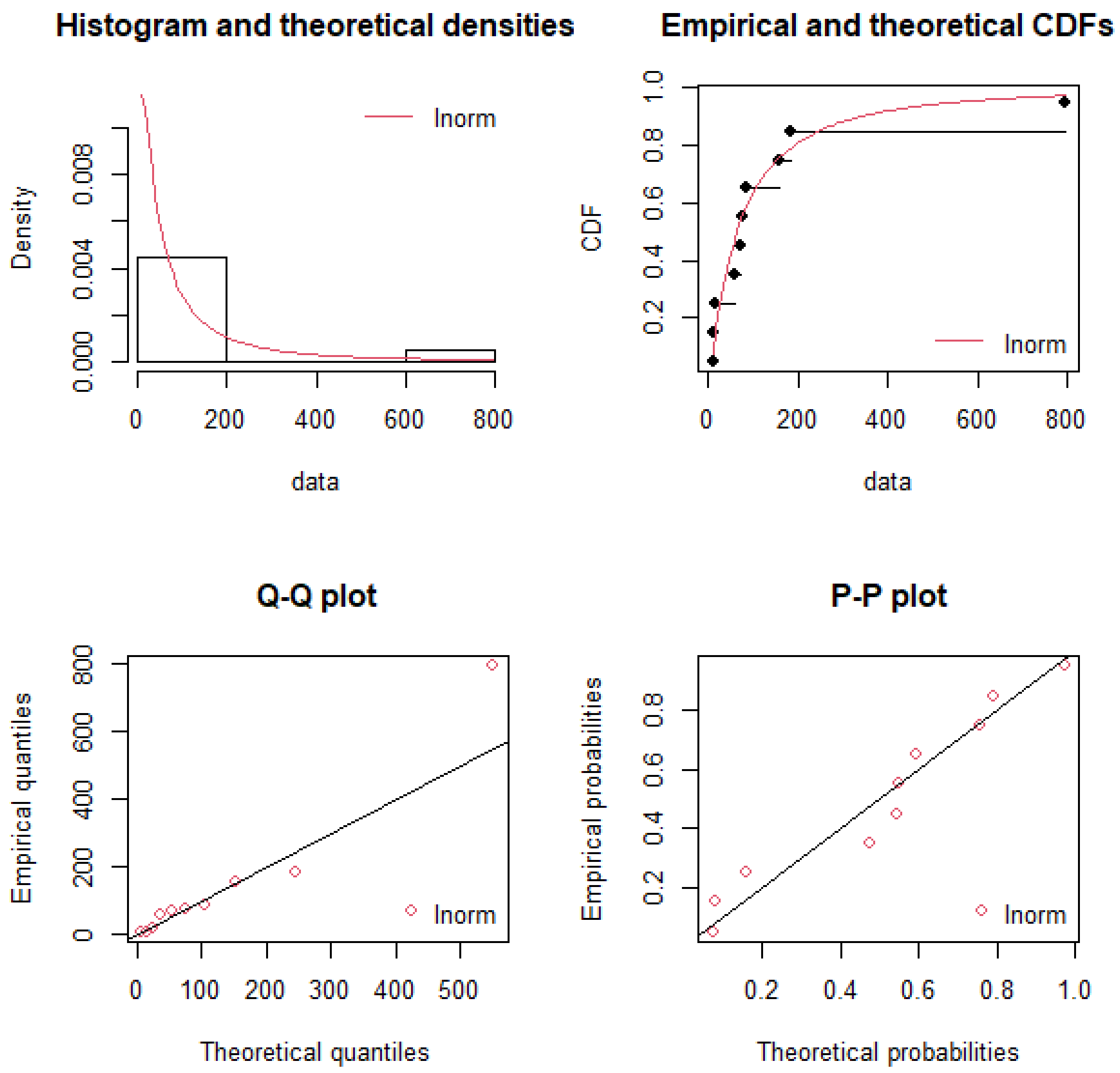

Figure S4: Log normal distribution fit to the previous user of tape data $\overline{RFU}_{C2tape}$.
